# How we learn social norms: a three-stage model for social norm learning

**DOI:** 10.3389/fpsyg.2023.1153809

**Published:** 2023-06-02

**Authors:** Wen Zhang, Yunhan Liu, Yixuan Dong, Wanna He, Shiming Yao, Ziqian Xu, Yan Mu

**Affiliations:** ^1^CAS Key Laboratory of Behavioral Science, Institute of Psychology, Chinese Academy of Sciences, Beijing, China; ^2^Department of Psychology, University of Chinese Academy of Sciences, Beijing, China; ^3^School of Humanities and Social Science, Chinese University of Hong Kong, Shenzhen, China; ^4^Faculty of Education, Beijing Normal University, Beijing, China; ^5^Department of Psychology and Behavioral Sciences, Zhejiang University, Hangzhou, China; ^6^Graziadio Business School of Business and Management, Pepperdine University, Los Angeles, CA, United States

**Keywords:** social norm, learning process, reinforcement learning, culture, neural mechanisms

## Abstract

As social animals, humans are unique to make the world function well by developing, maintaining, and enforcing social norms. As a prerequisite among these norm-related processes, learning social norms can act as a basis that helps us quickly coordinate with others, which is beneficial to social inclusion when people enter into a new environment or experience certain sociocultural changes. Given the positive effects of learning social norms on social order and sociocultural adaptability in daily life, there is an urgent need to understand the underlying mechanisms of social norm learning. In this article, we review a set of works regarding social norms and highlight the specificity of social norm learning. We then propose an integrated model of social norm learning containing three stages, i.e., pre-learning, reinforcement learning, and internalization, map a potential brain network in processing social norm learning, and further discuss the potential influencing factors that modulate social norm learning. Finally, we outline a couple of future directions along this line, including theoretical (i.e., societal and individual differences in social norm learning), methodological (i.e., longitudinal research, experimental methods, neuroimaging studies), and practical issues.

## 1. Introduction

In the preceding decades, human societies have experienced dramatic sociocultural changes influencing human culture and psychology with globalization (Cai et al., 2019). Social norms are widely viewed as the common values, expectations, and beliefs shared by most members of the group and society (Elster, 1989; Hechter and Opp, 2001). To maintain and organize a stable and sustainable society, humans have developed and enforced a wide range of social norms, which are of significance in guiding individual and group behaviors at the micro-level (Smith and Louis, 2009; Liu et al., 2018; Gilliam et al., 2022) and promoting social order and large-scale cooperation at the macro-level (Morris et al., 2015; Mu et al., 2015; Gelfand et al., 2017; Legros and Cislaghi, 2020). Generally, social norms are viewed as the unique glue of human societies, as humans conform to social norms to fulfill the mutual expectations within the social group (Chudek and Henrich, 2011; Schmidt and Tomasello, 2012). However, when entering into a new situation or culture, people are usually exposed to a variety of unfamiliar, ambiguous, implicit rules and norms, most of which are unwritten and situation-dependent social norms. Thus, understanding the potential mechanisms of processing these new social norms provides an insight into how culture and its changes may influence people’s minds and behaviors (Gelfand et al., 2011; Morris et al., 2015; Chua et al., 2019) and how people could better learn and adapt to a fresh cultural context.

Most of the time, people feel obligated to conform to a certain social norm (e.g., a group or the majority’s opinion) even when they know it is wrong (Asch, 1951), which is related to the specific features of social norms. Different from other types of norms (i.e., personal norms, moral norms) based on internal values (e.g., morality, virtue), social norms contain stronger sociality and sustaining motivation because they can hardly be learned, maintained, and adapted without social contexts and feedback (Bicchieri, 2006; Morris et al., 2015). As a basis of other norm-related processes, accumulating evidence has revealed the mechanisms of various norm-related processes, including norm conformity (Hodges, 2014), norm enforcement (Horne, 2007), and norm violation detection (Mu et al., 2015; Van Kleef et al., 2015), yet limited attention has been paid to the possible mechanisms of individuals’ learning new social norms.

Previous studies have found that norm differences vary across even within the country (Harrington and Gelfand, 2014; Chua et al., 2019), depending on sociocultural factors such as environmental threats, rice-farming, and urbanization (Chua et al., 2019; Talhelm and English, 2020). For example, people who move to a larger and more developed city may need to sort and put garbage in a specific place to effectively improve the environment and promote resource recycling. Considering these cultural differences (e.g., urbanization) is inevitable, for an individual, a higher ability to learn social norms in a novel culture is an absolute and urgent necessity to respond to such urbanization-related norm changes and acculturation more quickly and adaptatively. Moreover, for society, learning social norms about devotion or diligence is advantageous for cooperation (Schmidt and Tomasello, 2012; Anderson and Dunning, 2014; Dannals and Miller, 2017; Legros and Cislaghi, 2020). Although complying with social norms is not always beneficial for individuals, like youth smoking uptake (East et al., 2021) and feuding behaviors (Young, 2015), the ability to learn social norms is necessary and can serve as a requisite for other related processes, ensuring people follow the majority (Anderson and Dunning, 2014; Olsson et al., 2020). Given the broad implications of social norms and their benefits in understanding individual adaptation and social coordination to cultural changes, especially when entering into a new environment or experiencing sociocultural changes, there is an urgent need to focus on the mechanisms underlying social norm learning.

Considering the significance of learning new social norms on sociocultural adaption to a changeable environment and a fresh cultural context, we review previous literature on social norms and different types of learning to provide a potential theoretic framework for social norm learning. We first start with distinguishing the unique features and the significance of social norms from other types of norms. Second, for each of the specific stages, we propose an integrated model of social norm learning, which lay the foundation for further research on social norm learning at the individual level. Third, we additionally highlight the potential brain regions that may involve in support for social norm learning at the neural level and further discuss individual and social moderating factors that may influence social norm learning. To spur future research on the process of social norm learning, we conclude with a discussion of exciting frontiers that we envision.

## 2. The features of social norm

When people come into a fresh situation or culture, social norms are usually unwritten and implicitly reflect how people should behave in the group or society of a certain situation and culture. Although different types of norms share the same core—behavioral patterns in groups or “group-level evaluations of behavior” (Horne and Mollborn, 2020), unwritten social norms in a new situation or culture are unique in multiple aspects.

One of the key features making social norms different from other types of norms (e.g., conventions, personal norms, moral norms, and legal norms) is sociality. Mackie et al. (2015), by comparing social norms with several types of norms, viewed social norms as “strongly social,” which reflects one’s perception of others’ expectations about what should be done in a certain situation. Thus, compared to following conventions which is usually driven by self-interest and sustained by empirical expectations, people comply with social norms to even be at odds with their personal goals and maintain them by normative expectations (Bicchieri and Muldoon, 2014; Gross and Vostroknutov, 2022). On the contrary, personal norms are internalized values that are classified as “not social” and call for less social pressure to maintain (Bicchieri, 2006). Further, although moral norms can be shaped by societal factors, social norms are primarily driven by social motivations, necessitate conditional preferences for compliance, and reflect social pressure acknowledged through social feedback in specific situations (e.g., rebuke, gossip, stopping one’s behavior, approval, or compliment; Bicchieri, 2006; Morris et al., 2015). However, the sources of social feedback are diverse. Different from the feedback of legal norms which are more formal and commanded by institutions and special groups (e.g., the police; Mackie et al., 2015), the reference group of social norms changes with different contexts and social interactions, including all relevant others whose behaviors and opinions matter, such as peers, friends, and family (Legros and Cislaghi, 2020). That is, learning social norms relies on social interactions with different groups, which underscores the sociality of social norms.

The second prominent feature of social norms is situation-dependent or situation-sensitive. Unlike moral norms, social norms are not fundamentally right or wrong and are more dependent on situations. Thus, the new social norm we learn in a particular situation may not apply to another case. Take playing music as an example, you will not be accused of doing so at the city square where people regard it as the appropriate behavior, while this may not apply to a back road where people are expected to be quiet. On the contrary, personal and moral norms, guided by beliefs from long-term experience, are less influenced by the situation (Anderson and Dunning, 2014). For instance, a person who has the moral belief of “not to lie” will not lie even in a situation where lying is common and never be punished. Compared to legal norms reflecting the internalization of institutions, the most fundamental criterion of social norms determining what people should do remains situation-dependent (Morris et al., 2015): the norm of “no jaywalking” does not exist in a chaotic street where everyone does so.

## 3. Social norm learning and its unique “social” features

When coming into a new situation or culture, learners need to interact with others to be ready for learning new social norms in this circumstance, where social interaction are of importance in newcomer learning (Korte, 2009). In comparison to other learning processes (e.g., general reinforcement learning, learning of moral norms), social norm learning may have its uniqueness inherited from the key features of the social norms concerned, which embodies three “social” characteristics from individual to group level: social cognition, social feedback, and social context.

First, social norm learning requires social cognition, such as sharing and understanding others’ mental states and behaviors (Legros and Cislaghi, 2020), reflecting learners’ ability to interact with others. Regarding social norm learning, less is achieved strictly from personal experience, whereas more is acquired from relevant others (Bossan et al., 2015). These processes are often considered to include social learning (i.e., learning from others). Thus, the mechanism of social norm learning resembles two social learning strategies. One is called the frequency-dependent strategy by which people will adopt the action that is the most common in a reference group, and the other is defined as the pay-off-based strategy, which highlights that the selection of a certain behavior depends on the feedback from the observed others (Morgan et al., 2012). That is, learners acquire information about social norms from social interactions with others as well as the preliminary knowledge of social norms that people pass on (Mackie et al., 2015). Although perceived behaviors and beliefs of the majority are the two main sources of social norm predictions (Cialdini et al., 1991), not all evidence is treated equally during the process of evaluation, which may make different learners host different sensitivity to others’ behaviors and cause them to assume that some people are more reliable than others in turn. In other words, social norm learning relies heavily on social cognition, i.e., judgments about the reliability of others’ behavior (Muldoon et al., 2014).

The second key feature of social norm learning is social feedback, which supplies learners’ prediction of appropriate behaviors when interacting with others. Paying attention to what most people do (i.e., descriptive norms) could help collect the initial information and further prompt subjective expectations of social norms (Morris et al., 2015). On the one hand, the learning mechanism could function via social reference, that is, people learn to behave appropriately in a certain situation based on the observation of others’ behaviors and the feedback received. More importantly, the social feedback an individual received from others plays a direct role in strengthening or weakening one’s estimation of the appropriateness of behavior in a certain situation, which includes various types of social feedback, such as physical actions, emotional reactions, and marking schemes (expressed by adding or deducting points; Fix et al., 2006; Morris et al., 2019). For instance, one can learn not to make noise in the library by having been reprimanded or learn to offer seats to the elderly by having been praised. Thus, not only can individuals behave as the norm asked by observing others’ shared experiences, but they also can experience the social feedback on their own more directly.

Finally, given that social norms are sensitive to sociocultural contexts, the third key characteristic of social norm learning is context-based processing, which makes learning social norms different from other learning processes, such as reinforcement learning depending on the feedback valence and strength (Klucharev et al., 2009). Additionally, unlike moral beliefs requiring less strengthening and would hardly fade out in changeable situations due to internalization (FeldmanHall and Dunsmoor, 2019), social norms can be learned through personal experiences/attitudes and be acquired from the surrounding environment and the potential reference groups (Muldoon et al., 2014). Therefore, the learning process of social norms is highly relevant to the ongoing circumstance and context, which helps learners to detect the new social norms when interacting with others.

## 4. The three-stage model of social norm learning

Imagine when you leave your hometown for a new city to work and live in, and you would notice various new social norms to learn and adapt to, such as no littering and garbage classification. To timely and adaptively respond to this circumstance, you may first observe and collect some social and environmental cues about the new social norm in this situation. Then, you may generate a prediction about how to behave appropriately in this case, and the social feedback you received (e.g., others’ smiling in approval or shaking heads in disapproval) may help to adjust your prediction. By continuously adjusting your prediction based on the feedback, you will learn this new social norm and behave appropriately next time in this café.

**Figure 1:**
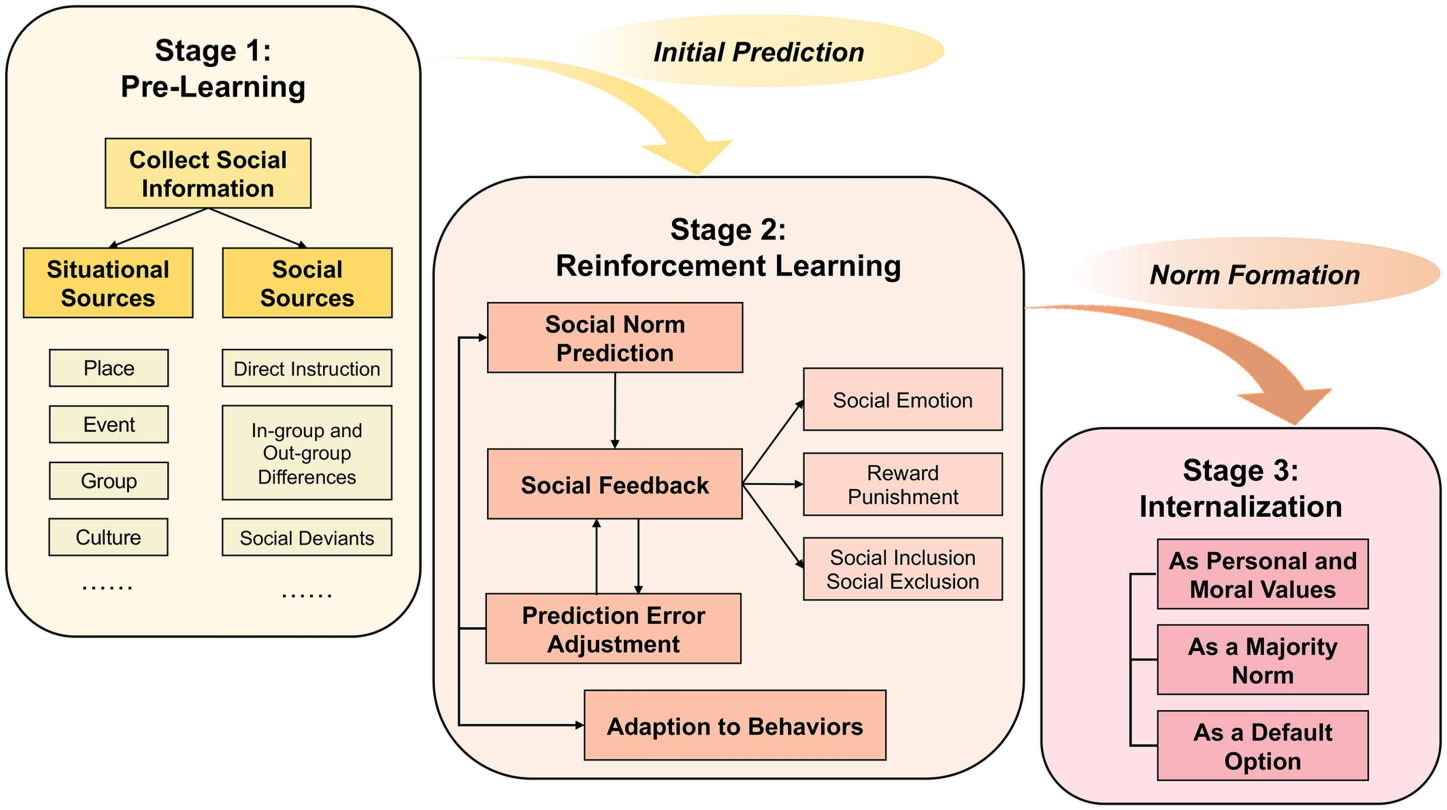
The three-stage model of social norm learning, as a step-by-step, dynamic, and cyclic process of learning a new social norm in a certain situation or new cultural context, includes three stages: **(1)** Pre-learning stage is referred to the process that individuals perceive the social norms through the collection of various social information in certain situations from situational sources (e.g., the place where a social norm happens, the present group which learner belongs to) and social sources (e.g., differences between the new norm in in-group and for outgroup, social deviants), which is useful to make people realize that there may be the new social norm in such situation and form the initial social norm prediction of this new social norm; **(2)** Reinforcement learning stage is a dynamic process of forming various predictions of this new social norm, receiving social feedbacks from others (e.g., social emotion, reward and punishment, social inclusion, and exclusion), adjusting the prediction error and forming a new prediction, and finally form the concise and appropriate prediction of this new social norm to adapt to others’ behaviors; **(3)** Internalization stage aims to integrate into new social norms and extend the former norm system, which is involved in three ways for those who regard this new social norm as personal and moral values, a majority norm, and a default option.

After the in-depth view of the key features of social norm learning, we propose that learning a new social norm embodies three stages in this section: (1) collecting information or cues embedded in the interactional situation (*the pre-learning stage*); (2) dynamically learning how to behave correctly by receiving social feedback and adjusting prediction error (*the reinforcement learning stage*); and (3) internalizing the new social norm into the norm system (*the internalization stage*; see Figure 1).

### 4.1. Stage 1: pre-learning

The initial period of learning new social norms, namely the pre-learning stage, is mainly activated to help people collect social information and get ready to form an initial prediction. Due to the desire for norm conformity to generate positive emotions (i.e., feeling good and energetic) and social identification (Christensen et al., 2004), learners will try to accumulate various sources of social information. When interacting with others in a new cultural context or a temporary situation where they need to detect whether there is a certain normative behavior to follow, learners will pay attention to others’ reactions and monitor themselves to behave as appropriately as possible (Muldoon et al., 2014).

Therefore, in this stage, one of the most significant processes is *norm detection*, which refers to a dynamic norm-learning process of discovering the potential norms in a certain situation by paying attention to and collecting information and cues from situational and social sources when learners observe and interact with others (Mahmoud et al., 2014; Legros and Cislaghi, 2020). The situational sources mainly include place (where a social norm takes place), event (which devotes to a social norm), group (to which learners belong), and culture (which reflects macro-level contextual factors). As for the social sources, several cues in guiding social interaction should be emphasized, including direct instruction (e.g., education, signs and texts in the public area, and straightforward verbal norms), others’ emotional reactions and behaviors, in-group and out-group differences (i.e., differentiating the norms suitable for in-group from those for out-group), social deviants, etc.

As people need to collect social information and cues in the pre-learning stage, monitoring others and themselves is thought to be another important process. Learners could observe others’ actions to figure out the appropriate behavior in this social context, which means successfully monitoring others’ behaviors could help learners better acquire the knowledge of others, especially the prototypes which are regarded as the most typical examples (Snyder and Cantor, 1979). Meanwhile, monitoring one’s own behavior is the key ability to ensure one behaves appropriately and avoid norm violation (Snyder and Cantor, 1979). Active self-monitoring helps individuals form an appearance that caters to the current situation and obtain more positive rewards from others. An EEG study investigated the neural activation of action monitoring in psychopathy individuals and found that psychopathic relative to healthy people (the control group) elicited decreased error-related negativity (ERN, a neural marker of others’ incorrect behaviors) during the observation of others’ actions, which might hinder psychopathic participants’ social norm learning via observation (Brazil et al., 2011). Compared to the long process of collecting social information and monitoring others, there might be a fast process to help learners adapt to the new situation in the pre-learning stage. Specifically, the former knowledge of acquired social norms in a similar situation is beneficial to facilitate such fast processing of social norm learning. Sensitization of fast learning process may include but are not limited to transfer learning, school studying, and verbal injunctive norms. To sum up, the pre-learning stage is mainly devoted to generating the initial prediction of a new social norm by monitoring others and themselves to detect the behavioral pattern with the majority and then recognize it as the “norm.”

### 4.2. Stage 2: reinforcement learning

After information collection and norm detection in the pre-learning stage, learners have formed the initial prediction of the new social norm and then enter the next stage during which they interact with others in the given situation and receive social feedback, in turn, leading them to adjust and update their predictions of the new social norm. The second stage of social norm learning is a dynamic process of reinforcement learning. In previous literature, reinforcement learning is usually operationally defined as a trial-by-trial learning process in response to feedback to adjust the prediction for the correct behavior (Garrison et al., 2013; Cheong et al., 2017). Based on the process of reinforcement learning, we propose that the dynamic learning process contains three interacting components: *social norm prediction, social feedback, and prediction error adjustment.*

#### 4.2.1. Social norm prediction

Forming the primary and initial representation of a new social norm requires social information and sources in the pre-learning stage—what most people would do or may approve of in this certain situation or new cultural context. When there are enough demonstrations, learners can observe how others behave, receive relevant feedback, and combine them with endogenous signals (e.g., evaluation of the demonstrators’ behaviors) to adaptively imitate (Najar et al., 2020). If the information is insufficient, learners need to act by trial and error, which may rely on individual learning strategies (Bossan et al., 2015), including inferring the purpose, evaluating the reliability of the information based on previous observation, deducting different consequences, and behaving to best fulfill the purpose.

#### 4.2.2. Social feedback

Once learners behave according to their social norm prediction and act by trial and error to form the appropriate social norm, the adjustment of social norm predictions can hardly achieve without social feedback, which has long been viewed as a pivotal driving force that leads to behavior change. Therefore, the generation and dissemination of social feedback by other people (i.e., an ingroup member) are an important basis for reinforcing and consolidating social norms. Learners can obtain social feedback not only on their own behaviors but also on that of others so that they can reason what most group members approve or disapprove of and pave the way for adjusting the social norm prediction. At the same time, learners’ behaviors may become observers’ social feedback from others. As a result, the shared behavioral pattern of most members of a group or society generates and ripples through within the group. This mechanism would even work without specific guidance or written rules because people treat each other as their reference points. The network trains itself via the distributed but shared psychological processes of the group or society members.

Except for different sources of social feedback, there are many forms of social feedback, whether it is social identity, reward value, approval of others, punishment for violating norms, or psychologically painful experiences like social exclusion (e.g., Klucharev et al., 2009; Rudert and Greifeneder, 2016; Halmesvaara et al., 2020; Molho et al., 2020). Emotion is one of the most powerful social feedback when it comes to helping people quickly adapt to the situation. For instance, emotional signals (i.e., sadness, anger, and shame) help people effectively detect a norm transgression behavior (Hareli et al., 2013), an attitude change during social interaction (Morris and Keltner, 2000), and the recognition and prevention for a norm violating behavior (Halmesvaara et al., 2020). In addition, norm-related emotions like shame or guilt of the reference group could trigger painful experiences of norm transgression, which prevents people from behaving inappropriately (Rudert and Greifeneder, 2016). In other words, influential and proper social feedback will accelerate the adjustment for prediction errors.

#### 4.2.3. Prediction error adjustment

After receiving feedback, learners need to solve the prediction errors—the differences between their expectations (i.e., the previous prediction) and the obtained consequences or feedback (Schultz and Dickinson, 2000; Garrison et al., 2013). Moderate prediction errors are beneficial to learners’ timely adjustment of appropriate behaviors and then facilitate the process of learning new social norms, while high prediction errors might lead to learners’ cognitive conflicts toward their prediction and then hinders their acquisition of new social norms. Generally, learners first keep the differences in mind, modify false beliefs in time, adjust their predictions, and finally adapt to appropriate behaviors (Cialdini and Goldstein, 2004). Berns et al. (2010) found that individuals tended to adjust their ratings to be consistent with group preference to relieve anxiety. Using advanced computational modeling (i.e., the Bayesian learner model), researchers further describe the adjustment process in detail that learners first observe the preference of the majority from others’ behaviors, integrate their preference (transcendental belief) with the preference of the majority (social norm) by updating (Bayesian) belief, and then make their behaviors adapt to the group social norm (Muldoon et al., 2014; Garvert et al., 2015; Suzuki et al., 2016; Reiter et al., 2019). As a result, prediction error adjustment plays a crucial role in forming the representation of a new social norm.

### 4.3. Stage 3: internalization

Internalization is a key element of socialization whereby one follows a certain social norm from one’s own internalized motive rather than out of external surveillance or sanction (Etzioni, 2000; Bell and Cox, 2015; Morris et al., 2015; Dannals and Miller, 2017). That is, people follow the new social norm even when others around them do not obey it after internalizing it. However, how a new social norm can be stored and represented internally remains unclear. We propose three potential ways through which a new norm can be internalized. First, for people who regard this social norm as their values (Anderson, 2000; Bell and Cox, 2015), the new social norm might be integrated into the previous system related to their personal and moral values. Second, a new social norm can be viewed as a representation of what “the majority” does. Therefore, people who tend to comply with “the majority” to obtain their identities from society or the organization may internalize the new social norm as a majority norm (vs. minority norm). Third, a new social norm can be cached as a default option. When facing a lack of alternatives or having been exposed to uncertain situations, people may activate the default option to follow this new norm temporarily. Although simple norm conformity does not necessarily need internalizing the norm, once people internalize the norm, they not only comply with it but also show emotional reactions reflecting norm enforcement when facing a norm-violation behavior. Previous research has found that emotions facilitate norm enforcement in a third-party punishment task (Fehr and Fischbacher, 2004; Nelissen and Zeelenberg, 2009; Pfattheicher et al., 2019), which suggests a potential role of emotion in norm internalization (Gross and Vostroknutov, 2022). In summary, the internalization of new social norms is crucial for maintaining this norm, detecting norm violators, and complying with or enforcing this norm in certain situations. In the long run, people would adapt to a new and changing culture better if they could internalize these new norms.

To conclude, the three-stage model devotes to the main process of social norm learning: (a) Pre-learning stage is aimed at collecting social information from two social sources in a certain situation, including situational sources and social sources, which devotes to forming the initial prediction of a new social norm while preparing for next stage; (b) Reinforcement learning stage includes the substages of forming the social norm prediction, receiving social feedback from others, and continuously adjusting the prediction error between the previous prediction and the outcome, which is a cyclic and dynamic process that aids in updating the primary representation, adapting to others’ behaviors, and eventually forming the new social norm; and (c) Internalization stage includes three possible ways for those who regard the new social norm as personal and moral values, a majority norm, and a default option, respectively, to incorporate the new social norms into the former norm system. This is a step-by-step process of this three-stage social norm learning model from forming the initial social norm prediction to continuously adjusting the prediction error dynamically and internalizing the final social norm into the norm system to help one behave appropriately in this situation in the future (see Figure 1).

## 5. Neural mechanisms of social norm learning

Taking advantage of functional magnetic resonance imaging (fMRI), previous literature has mapped distributed brain networks involved in social norm-related processing, such as social norm compliance (Spitzer et al., 2007; Ruff et al., 2013; Makwana et al., 2015), social learning (Rilling and Sanfey, 2011; Joiner et al., 2017), and reinforcement learning (Behrens et al., 2008; Apps et al., 2013, 2015). However, limited attention has been paid to the social norm learning process and its neural substrates. In this section, by reviewing relative studies in this field, we propose the potential neural circuits of social norm learning, especially for the requisite processes (i.e., the pre-learning stage, and the reinforcement learning stage) during which a social norm is learned.

As we mentioned, pre-learning is to generally detect and collect social information from the current situation and monitor one’s behavior. This process demands the involvement of the “social brain”—brain regions (e.g., the frontal cortex) that are evoked by the mere presence of social information (Tso et al., 2018). For instance, the dorsal medial prefrontal cortex (dmPFC) is engaged in perceiving complex social stimuli (Wagner et al., 2016), integrating social information (De Martino et al., 2017), and performing social reasoning (Fiddick et al., 2005). Specifically, during the pre-learning stage, learners will generate their representation of appropriate behavior in the current situation (i.e., social norm representation) which is highly associated with the right medial frontal gyrus (MFG; Zinchenko and Arsalidou, 2018)—a region involved in processing one’s expectation and inference of existing norms (Christoff et al., 2009). With the social norm being represented mentally, individuals may need to monitor their behaviors to comply with the norm. It has been suggested that patients with lesions in the orbitofrontal cortex failed to monitor themselves even though they know what the norm is (Beer et al., 2006). This phenomenon is highly attributed to the incompetence of monitoring social feedback from others caused by orbitofrontal damage (Kringelbach and Rolls, 2004). We, therefore, speculate that the prominent role of the orbitofrontal cortex in facilitating self-monitoring of norm-compliance behavior.

Different from the pre-learning stage, the reinforcement learning stage highly relies on how learners utilize social feedback (i.e., reward or punishment) to reinforce and adjust their predictions and behaviors. How does the brain process social feedback? Previous literature on positive feedback has shown that receiving and anticipating positive social feedback, like smiling faces and verbal praise, are likely to induce greater activation in the ventral striatum (*VS*), particularly in the nucleus accumbens (NAc; Kirsch et al., 2003; Spreckelmeyer et al., 2009; Rademacher et al., 2010). On the other hand, negative feedback, such as social sanctions, promote norm compliance by modulating the right lateral prefrontal cortex (Ruff et al., 2013). It has been documented that the ventral anterior cingulate cortex (vACC) is particularly sensitive to social feedback rather than expectancy violations (Somerville et al., 2006), suggesting that the vACC plays a vital role in processing the feedback in social norm learning. However, researchers also found that the dorsal ACC (dACC) is responsive to expectancy violations (Somerville et al., 2006), which suggests that when learners receive feedback, their estimation of prediction error (i.e., the discrepancy between an expected outcome and what happens) may be associated with the ACC (Schultz and Dickinson, 2000; Garrison et al., 2013). In addition to rewards and sanctions directly related to oneself, value and prediction errors of others’ behavior can also reinforce learners’ knowledge of social norms. The gyral surface of the anterior cingulate (gACC) is specifically responsible for learning others’ prediction errors (Apps et al., 2012, 2013), which can be regarded as part of observational learning (Hill et al., 2016).

Taken together, the current research has mapped a potential brain network in processing social norm learning, with the medial prefrontal and the orbitofrontal cortex involved in the pre-learning stage, and the brain network related to social feedback (e.g., the ventral striatum, ACC) engaged in the reinforcement learning stage.

## 6. The influencing factors of social norm learning

While people have been universally equipped with the ability to learn social norms, it is no doubt that this ability varies across individuals and groups. For example, cross-cultural evidence showed that cultural tightness predicts higher sensitivity to norm-violation behavior (Mu et al., 2015). In this section, we first discuss the cultural dimension based on norm strength, namely *cultural tightness–looseness* (TL), highlight the effect of other cultural variations (i.e., individualism–collectivism, the rice theory), and then point out a set of individual factors that may impact social norm learning.

### 6.1. Cultural factors

Though social norms are universally established in all human groups and societies, cultural variations in the strength of social norms and the degree of tolerance for norm deviance have been deeply discussed in a multilevel framework of cultural TL. Such cultural variations are predicted by the degree of ecological and historical threats that different societies have experienced (Gelfand et al., 2011), not only between countries but also within themselves (Gelfand et al., 2011; Harrington and Gelfand, 2014; Chua et al., 2019). Specifically, tight societies have higher levels of historical threats (e.g., natural disasters, conflicts, and high population density), and thus they have developed stronger norms and harsher punishments for norm-violation behaviors (Gelfand et al., 2011). For individuals to detect norm-related cues, tight cultures have clearer norm instructions and stricter norm enforcement, and people in a tight society are cultivated to be more vigilant in seeking social norms and are high in self-monitoring. As a result, they may have more “felt accountability” for learning social norms. According to the cultural framework, it is speculated that cultural TL may modulate the pre-learning stage when people need to learn new social norms in the current situation or cultural context. Tight cultures impose more restrictions on people’s daily behaviors by providing more guidelines and regulations in public settings, such as the sign for no chewing gum in Singapore (Gelfand et al., 2011; Chua et al., 2019). Consistent with this, Mu et al. (2015), by using the electroencephalogram technique, found that people from a tight culture exhibited increased endorsement of inappropriateness and greater neural responses to norm violation behaviors. Furthermore, people from tight cultures (e.g., China, Japan) tend to monitor and regulate their behaviors in many domains (e.g., eating behavior, alcohol consumption, and emotional regulation), as compared to those from loose cultures (e.g., American) who exhibit more self-regulation failures (Gelfand, 2018). Accordingly, it is assumed that people from a tight culture are chronically exposed to stricter social norms, which is beneficial for them to detect social norms and monitor their own and others’ behaviors during the pre-learning stage.

Cultural TL may contribute to the sensitivity to social feedback and behavioral adjustment during the reinforcement learning stage. The tight culture societies have stronger punishment for norm-violation behaviors and social praise for the behaviors that obey norms (Gelfand et al., 2011), which is favorable to the individuals to form the norm prediction and make appropriate adjustments according to the social feedback constantly received in tight cultures (Thombs et al., 2007; Li and Yan, 2020). Moreover, people in a tight society who do not adhere to norms are more likely to be ostracized and excluded by other society members (Whitson et al., 2015) and are more vulnerable to social sanctions and punishment (Molho et al., 2020). That is, in a tighter culture, people tend to be more sensitive to social feedback and feel more accountable for adjusting their behaviors to reduce the risk of violating the social norm and being punished. Thus, as a norm-based cultural dimension, we propose that cultural TL will play an unignored role in shaping and modulating multiple key processes of learning social norms.

Except for the effect of cultural TL on social norm learning, other cultural variations may also influence newcomers to learn social norms. On the one hand, individualism–collectivism reflects the concern for self and others (Oyserman et al., 2002). Compare to individualism valuing the expression of individual independence and internal traits as the core, collectivist cultures emphasize compliance with obligations, compromise, and harmony (Triandis et al., 1988, 1990), and focus on common fate, goals, and values in the society (Oyserman et al., 2002), which may be related to norm-related processes, such as norm perception (Sherman et al., 2021) and attitudes toward norm violators (Stamkou et al., 2019). On the other hand, the rice theory indicates that people living in rice-growing areas tend to be more communal and have stronger social norms (Talhelm et al., 2014; Talhelm and English, 2020). Thus, stronger and tighter social norms in the rice regions may affect stronger punishment for violators and a higher desire to conform to social norms, which would help people to detect new social norms in the pre-learning stage and adjust their behaviors after receiving the feedback in the reinforcement learning stage.

### 6.2. Individual factors

Accumulating studies have indicated that people perceive, react to, and enforce social norms differently (Jacobson et al., 2015; Friehe and Schildberg-Hörisch, 2018; Xie et al., 2020), we thus speculate that individual factors related to norm processing may moderate social norm learning. First, the pre-learning stage discussed above requires sensitivity to processing social information embedded in a certain situation or a changing cultural context. Learners with high social sensitivity relative to those with low sensitivity are better at detecting subtle social cues (e.g., social experiences, brief conversational silences). We, therefore, conjecture that individual sensitivity to social information contributes to gathering and processing social cues, which further help behavioral adjustment to fulfill social expectations. Second, people with high self-monitors tend to behave appropriately to fit into a certain situation and change their behaviors when they are aware of changes in the situation, whereas low self-monitors rely more on their internal cues (Snyder, 1974; Smith et al., 2019). Thus, compared to low self-monitoring people, when those with high self-monitoring are exposed to cultural changes or experience temporary changes in a certain situation, they could detect external others’ behaviors more quickly to behave more appropriately, and finally, facilitate the pre-learning stage. Third, the individual ability of mentalization (i.e., the capacity of speculating about other people’s mental states and predict their behaviors; Frith and Frith, 2006), is closely related to norm-related processes, such as norm-enforcement (Baumgartner et al., 2012), sharing others’ norm transgression (Paulus et al., 2015), and social norm inferences (Pegado et al., 2018). This capacity could be strengthened via actively learning from others and dynamically updating predictions by combining various aspects of social information (Silston et al., 2018; Wu et al., 2020). Learners with high mentalizing capacity relative to those with low mentalization may have more advantages in understanding others’ behaviors and intentions, which helps them to interpret the new social norm and form a concrete prediction. Fourth, compared to the ability of mentalization showing one’s understanding of others, metacognition refers to the ability to represent, monitor, and control one’s mental cognitive processes (Norman et al., 2019; Heyes et al., 2020), which helps learners monitor or regulate the cognitive resources required in the reinforcement learning stage. It has been proven that people with high (vs. low) metacognition adjust better to a new country or city and learn new social norms comparatively faster (Klafehn et al., 2013; Shu et al., 2017; Morris et al., 2019). Moreover, metacognition-related processes (e.g., social judgments, representation of social knowledge or beliefs) are not just involved but also updated during social interactions like social acceptance or rejection (Petty et al., 2002, 2007; Frith, 2012; Wu et al., 2020). This suggests that metacognition may accelerate norm adjustment through accurately estimating prediction errors to form precise norm representation and make an optimal adjustment quickly.

To sum up, at the group level, in the cultural dimension, cultural tightness–looseness highlights group variations on the strength of social norms and the tolerance of norm violation, which further may impact how we initially form a norm prediction, process social feedback from others, and adjust to fit into different socio-cultural contexts. At the individual level, individuals’ social preferences and capacities, including social sensitivity, self-monitoring, mentalization, and metacognition, may facilitate the key processes of social norm learning when people face sociocultural changes.

## 7. Future directions

In this review, we have clarified the specific features of social norms and social norm learning, proposed a theoretical model of social norm learning, highlighted the neural mechanism of social norm learning, and discussed the cultural and social moderators. In this section, we further list a set of intriguing questions to be addressed in the future, including theoretical, methodological, and practical issues. In particular, we encourage future studies to (a) understand factors that influence the process of learning social norms, including contextual factors and individual differences, (b) need various methodological changes to explore the complex process of learning social norms, including longitudinal research, experimental methods, and neuroimaging studies, and (c) pay attention to practical application. The future directions are discussed below to deepen the understanding of social norm learning in multiple ways.

### 7.1. Societal and individual factors influencing social norm learning

#### 7.1.1. Societal influences

Although we focus on the individual-based model of learning social norms, various macro levels play a role in this learning process. At the group level, individuals observe and receive social information in certain situations to learn new social norms, while groups can teach, manipulate, and develop existing norms for newcomers (Korte, 2009). Moreover, newcomers may change previous norms and even introduce new ones (Harris et al., 2020; Otten et al., 2021). At the situational or contextual level, several factors (e.g., threat, risk perception, and uncertainty) be considered in future studies. For instance, certain situations with greater ecological threats (e.g., the COVID-19 pandemic) develop stronger social norms and punishments for violators to coordinate and maintain social order in face of these threats (Gelfand et al., 2017). At the cultural level, it has been well-documented that cultural values, norms, and practices shared by group members shape and are being shaped by a wide range of psychological and biological processes, such as social perception, emotion regulation, self-related thinking, mental attribution, etc. (Han and Northoff, 2008; Kim and Sasaki, 2014). Although empirical evidence has demonstrated cultural variations in norm-related processing (e.g., norm violation detection, and norm compliance; Gelfand et al., 2011; Mu et al., 2015; Salvador et al., 2020), little attention has been paid to the culture modulation on the social norm learning and its underlying mechanisms. At the ideological level, group conformity underlies social conservatism, promoting adherence to traditional social norms within a group and perceiving out-group threats and cultural boundaries (Claessens et al., 2020). Considering the importance of learning social norms for individuals navigating adaptive challenges during urbanization, we encourage future research to examine whether and how the changes caused by urbanization modulate the psychological and neurobiological mechanisms underlying social norm learning at the group, contextual, cultural, and ideological levels. Additionally, it is essential to explore how societal factors interact with personal traits/preferences to impact learning-related processes uniquely or jointly.

#### 7.1.2. Individual differences

Personal preferences as well as the individual factors that may help humans learn and internalize a new norm are urgently needed to be explored. As discussed above, high relative to low self-monitoring people are more responsive to adjusting their behaviors accordingly in different settings (Gomila and Paluck, 2020; Chen et al., 2021). However, little is known about the role of self-monitoring in modulating social norm learning. Future research is called to explore the mechanism by which self-monitoring affects the procedure and rate of social norm learning. Additionally, the sensitivity to social reward and sanction is particularly important for the reinforcement learning of social norms. Therefore, it is encouraged to test whether individuals with high sensitivity to negative feedback may induce more negative feelings and greater neural activation in the pain matrix when receiving negative social feedback from others (e.g., rejection, exclusion), as this may hinder the learning process.

### 7.2. Methodological changes in understanding social norm learning

#### 7.2.1. Longitudinal research designs

Considering the importance of social norm learning to human social development, such as children’s psychological well-being and social interaction in the future (Killen and Smetana, 2015; Tomasello, 2016), it is suggested that future studies may collect longitudinal data at different stages of childhood development to explore the developmental trajectory of social norm learning and identify causes and consequences of enhancing social norm learning and promoting social adjustment. Second, children could learn social norms from various interpersonal interactions, such as parent–child interactions (Nguyen et al., 2020) and peer interactions (Pinho et al., 2021). During middle childhood, positive mother–child interactions are important to children’s compliance behaviors (Zhao et al., 2021). Along with the growth of age, adolescents’ views of prosocial behaviors and social values are strongly influenced by peer norms (Pinho et al., 2021). Future studies are suggested to focus on the effect of different types of social interactions (e.g., interactions with parents, peers, or teachers) on children’s social norm learning in different stages of development. Besides, it is still unknown how social norm learning influences migrants’ psychological adjustment. Future studies could collect longitudinal data on migrants’ adjustment (e.g., depression, social anxiety, and loneliness) to explore the developmental trajectories of the ability to learn social norms and their socio-emotional adjustments.

#### 7.2.2. Experimental methods

Although there are some tasks designed to examine social-norm-related processing (e.g., norm detection; Mu et al., 2015; Salvador et al., 2020), they are not well suited for testing social norm learning due to the lack of manipulation of norm learning. Meanwhile, although accumulating attention has been paid to the understanding and mechanisms of the learning process (Klucharev et al., 2009; Morris et al., 2019), most of them have neglected social attributes (e.g., social contexts, social interaction, and social feedback) or the lack of manipulating norm learning. Thus, a pressing matter of future work is to develop a paradigm that integrates social norms into the learning process and allows learners to collect social information, receive social feedback, take actions, and adjust their behaviors to acquire a new social norm in a certain context. Expect for laboratory experimental research, more field experiments are encouraged to understand how social norm learning facilitates people to adaptively respond to the new cultural or situational changes in daily life.

#### 7.2.3. Advanced neuroimaging techniques

Although many efforts have been made on investigating social norms from the behavioral level, limited research using neuroimaging techniques uncovers social norm-related processes. To better pinpoint and track the dynamic changes of brain circuits involved in different stages of social norm learning, the high spatiotemporal resolution ma*gnetoencephalography* (MEG) technique is recommended to examine the network dynamics and features of social norm learning. This may help researchers to further understand the learning processes, i.e., from the detection of social cues in the pre-learning stage, to continuously adjusting behaviors according to social feedback, and internalization of the new norm. Noninvasive brain stimulation techniques, such as transcranial direct current stimulation (tDCS) and repetitive transcranial magnetic stimulation (TMS) allow researchers to further examine the dissociable causal roles of the brain regions involved in different stages of social norm learning.

### 7.3. Practical meaning of studying social norm learning

Ultimately, the new theoretical three-stage model we proposed in this review is beneficial for developing individual and group interventions. Growing evidence from cultural psychology has suggested that the migrants keeping the norms from their heritage culture (relative to the local norms) need to face opposition from native people (Karinen et al., 2019), and shared social norms between migrants and natives are helpful for migrants to form intergroup cultural integration (Choi et al., 2019). Furthermore, integration endorsement could improve the intergroup attitudes (e.g., British Muslims and White British, Abu-Rayya and Brown, 2023). However, limited studies have focused on the effect of social norm interventions on acculturation (i.e., adapting to a new cultural context). Previous cross-disciplinary studies used social norm interventions to solve specific problems in daily life, such as eco-protection (e.g., purchasing pro-environmental products; Kim and Seock, 2019). Thus, future studies could investigate which types of social norm interventions are effective in helping individuals learn novel social norms when entering a fresh cultural context to promote their socio-emotional adjustment and solve cultural conflicts. Specifically, various well-designed community or state interventions could be considered to promote movers’ adaptability to changeable cultural changes and even mitigate the cultural conflicts between native and migrant groups. For example, the community could supply local and migrant members with more social activities to help migrants understand new social norms and stimulate their energy to learn from other local members, and help natives understand foreign norms from migrants to reduce outgroup prejudice and discrimination and build friendly interpersonal relationship.

## 8. Conclusion

In sum, social norms play a critical role in the development of human society and individuals’ social development. No matter when individuals experience temporary situational changes or dynamic cultural changes, social norm learning is the indispensable process for individuals’ adaptively response to these sociocultural changes, and also the prerequisite process for norm enforcement, maintenance, and compliance. Different from other learning processes, social norm learning has its unique features, including social context, social cognition, and social feedback. In this article, we proposed that social norm learning includes three stages, i.e., pre-learning, reinforcement learning, and internalization. A series of environmental (i.e., cultural tightness) and individual factors (e.g., self-monitoring, social sensitivity, and metacognition) may modulate its underlying processes. Future directions are called for extending our knowledge and understanding at theoretical, methodological, and practical levels, from developing new paradigms to examining underlying causes and integrating them with frontier methodology and multidisciplinary approaches.

## Author contributions

WZ and YM conceived the review. WZ, YL, YD, WH, SY, and ZX performed the literature search. WZ, YL, YD, WH, SY, ZX, and YM supported in the drafting of the manuscript which was led by WZ and YM. WZ, YL, SY, and YM discussed the final model and edited the manuscript. All authors contributed to the article and approved the submitted version.

## Funding

This work was supported by the National Natural Science Foundation of China (32071016 and 32271129), CAS Key Laboratory of Behavioral Science, Institute of Psychology, Chinese Academy of Sciences (Projects 2019000050 and Y5CX052003), and the Scientific Foundation of Institute of Psychology, Chinese Academy of Sciences (Project E2CX3935CX).

## Conflict of interest

The authors declare that the research was conducted in the absence of any commercial or financial relationships that could be construed as a potential conflict of interest.

## Publisher’s note

All claims expressed in this article are solely those of the authors and do not necessarily represent those of their affiliated organizations, or those of the publisher, the editors and the reviewers. Any product that may be evaluated in this article, or claim that may be made by its manufacturer, is not guaranteed or endorsed by the publisher.
